# Needs for Technology-Enhanced Health Professions Education in Eastern and Southern Africa: Protocol for a Descriptive, Cross-Sectional Survey

**DOI:** 10.2196/67331

**Published:** 2025-09-02

**Authors:** Shalote Rudo Chipamaunga, Detlef Richard Prozesky, Elliot Kafumukache, Patricia Katowa-Mukwato, Kefalotse S Dithole, Lynette J van der Merwe, Mike Nantamu Kagawa, Rudo Gwini

**Affiliations:** 1 Department of Health Professions Education and Student Support University of Zimbabwe Harare Zimbabwe; 2 Department of Medical Education Faculty of Medicine University of Botswana Gaborone Botswana; 3 School of Medicine Faculty of Medicine University of Zambia Lusaka Zambia; 4 School of Nursing Sciences University of Zambia Lusaka Zambia; 5 School of Nursing Faculty of Health Sciences University of Botswana Gaborone Botswana; 6 Division Health Sciences Education University of the Free State Bloemfontein South Africa; 7 Department of Obstetrics Makerere University Kampala Uganda; 8 Department of Medicine National University of Science and Technology Bulawayo Zimbabwe

**Keywords:** education 5.0, substitution, augmentation, modification, redefinition, SAMR model, technology-enhanced health professions education, cross-sectional survey

## Abstract

**Background:**

The use of technology in its various forms has long been a feature of the education and training of health professionals in the industrialized world. As a result of the COVID-19 pandemic, health professions education institutions suddenly adopted “emergency remote teaching,” and this experience exposed the vulnerabilities of countries in Eastern and Southern Africa regarding modes of teaching and learning. In this region, the needs to migrate to effective technology-enhanced learning are not explicit.

**Objective:**

The main objective of this study is to assess the needs for technology-enhanced health professions education in Eastern and Southern Africa. This will lead to the development of a tool reflecting different technologies used in health professions education. The tool will be used by educators to identify and bridge gaps in their use of technology in health professions education.

**Methods:**

This will be a descriptive, cross-sectional survey, and data will be collected from medical and nursing programs at the bachelor’s degree level recognized by national professional bodies or government structures offered at tertiary institutions in countries in Eastern and Southern Africa. The substitution, augmentation, modification, redefinition (SAMR) model underpins our study and serves as an organizing framework for the different types of technology in current use in the institutions under study. The SAMR model is a tool that provides guidance in describing and categorizing uses of educational technology in the classroom. The model is intended to guide educators to enhance their teaching and learning through the adoption, adaptation, or transformation of educational approaches using technology. To obtain the purposive sample, a person from each program who is well-acquainted with the program will identify staff members and students who represent the totality of those populations fairly. Quantitative data were analyzed descriptively for each program. Data were then organized according to the SAMR framework to portray the types of technology in use and challenges encountered.

**Results:**

This research was funded in October 2023, and the first institutional review board approval was obtained in April 2024. Data collection began in September 2024 and ended in November 2024. Since this was a multi-institution study, we envisaged multiphase data analysis, which was completed in mid-December 2024. As of August 2025, the manuscript is under peer review for publication of the results.

**Conclusions:**

The study will reveal gaps in the use of technology, and this will lead to the identification of needs for enhancing technology in health professions education.

**International Registered Report Identifier (IRRID):**

DERR1-10.2196/67331

## Introduction

Education is an integral pillar of any country’s sustainable growth and development [1]. Higher education institutions are essential to a sustainable society’s growth and development and should function as transforming forces [2,3]. Regarding Sustainable Development Goals, Kasinathan et al [4] observed that technology has significant potential to drive humanity into sustainable development. The world also now finds itself in the fourth industrial revolution, also called Industry 4.0 [5]. As has been the case with the previous industrial revolutions, education is changing to accommodate the needs of the new revolution. This new system or set of educational processes has been termed Education 4.0. It aims to develop the competencies and skills that workers in the fourth industrial revolution require through applying active learning, technology-based learning, and student-centered learning [6,7].

More recently, Education 5.0 has arisen as a new concept [8]. It also involves the use of new technologies to transform education, but additionally emphasizes the need for a constructivist, humanistic, and andragogical orientation poised for deep learning [4,9,10].

Some higher education institutions have been influenced by the Education 5.0 movement, which is highly rated in terms of its association with current business needs and integration with industrial and technological developments [11,12]. Education 5.0 functions within Society 5.0, which is regarded as a super-intelligent society that promotes the convergence of cyberspace and physical space and focuses on human-oriented solutions and social innovation [3,13,14]. The expectation of such a combination is to develop an environment where humans and robots with artificial intelligence coexist and work to improve the quality of human life. A further development in the field of technology for education is the advent of disruptive technology like artificial intelligence software and social media, which are already being used by teachers and students worldwide [15]. Rowan and Casey [16] suggest a triple helix “academia-industry-authority” approach to creating and sharing disruptive tools for addressing emerging needs for development. Advances in industrialization require technology to enable adaptation to complex systems in the health sciences.

Against this background, the use of technology in its various forms has long been a feature of the education and training of health professionals [1,17]. Worldwide, a plethora of opportunities for using technology in such training have been developed and used, including augmented and virtual reality platforms that are available for use in modern education [18]. In clinical education, the process of acquiring knowledge and skills needs to be more experiential, self-directed, and hands-on than in many other disciplines. However, virtual or mixed reality interventions are becoming more accessible and can be used in an integrated way with current, more traditional learning systems [19]. Rath et al [20] point out that augmented reality and virtual reality technologies are already having a major influence on medicine and health care, the implication being that students need to be exposed to important skills in these areas.

Even before the COVID-19 pandemic struck, those institutions that had not taken serious measures to migrate to a higher level of use of technology would have been aware that they were lagging behind the requirements of modern educational approaches. In the situation created by the pandemic, students were unable to attend their usual learning events and yet had to move forward in their training. There is good evidence that the advent of large-scale national lockdown policies resulting from the COVID-19 pandemic stimulated the development of technology-enhanced health professions education (TEHPE) [21,22]. However, a study by Jeffries et al [23] found that while COVID-19 resulted in a transition to more web-based health professions education (HPE), the change was subsequently unstable since the change to technology was not always widely accepted, was marred by inequities (socioeconomic, gender, and geographical), and was not subject to ongoing evaluation to indicate where improvements were needed. Besides the lack of opportunities for essential learning in practical settings, the large-scale use of web-based learning during the pandemic also had ethical consequences—education with basic humanity was neglected and professionalism, interpersonal relationships, mentoring, and communication skills suffered. This trend may be continuing [24].

Challenges to expanding the use of technology in educating health professionals have also been discussed. Challenges such as unavailability, a lack of skills leading to resistance, and a lack of support that curtails the adoption and use of technology have been identified and some solutions have been outlined [17,25]. While acknowledging the empowering possibility of technology use in education in Africa, Cole [26] stresses the reality of the digital divide between and within countries and relating to gender. There is often a lack of connectivity, computers and other devices (let alone the more sophisticated technologies listed in the previous paragraph), and digital literacy. Even electricity supply has been and may still be a challenge [27]. An in-depth investigation of e-learning in sub-Saharan Africa and low-resource settings by Barteit et al [28] makes for sobering reading. They conclude that e-learning for medical education was not doing justice to medical students in these settings; that short, one-off projects were common (so-called “cul-de-sac pilots”); and that “technological development has overwhelmed rather than revolutionized medical education.” Both Cole [26] and Barteit et al [28] stress the need for sustained investment in integrating technology into existing platforms. There are also legitimate doubts about the effectiveness of the use of technology in training health professionals. In a large-scale Indian study by Hirkani and Supe [29], the evidence was mixed even though students had a positive attitude towards it. The authors concluded that more evidence needs to be generated regarding its effectiveness so that future decisions can be based on evidence. In Pakistan, Sadiq et al [30] report a broadly successful project to empower educators in incorporating greater use of technology in their teaching, but also noted educators’ limited e-learning skills and failure to engage with the new technologies, as well as technical hurdles encountered. Granger et al [31] have also found that the grand vision of Education 4.0 is in danger of being watered down by technology mostly being used for lower-level pedagogical changes (substitution and augmentation) in the substitution, augmentation, modification, redefinition (SAMR) model [32]. In this regard a study from Uganda revealed low uptake of TEHPE activities after 10 years of effort, again with lower-level pedagogical changes. The empirical evidence gathered in the study led to recommendations to create an e-learning unit, to improve skills and knowledge in educational technologies, to expand infrastructure for TEHPE, and to design and implement an educational technology policy [33].

From the available evidence reviewed, there is clear evidence that TEHPE is thoroughly in place in the world, and that this trend is likely to have intensified during the COVID-19 pandemic. Interestingly, however, there is little information available about the extent to which TEHPE is currently being used in the training of health professionals specifically in Eastern and Southern Africa. It is also not known whether the sudden spurt in TEHPE during the COVID-19 pandemic has been maintained, nor is it known whether what has been retained is of a quality that deserves its retention. The obstacles impeding its use and the factors facilitating its use in this geographically specific setting are similarly not known.

**Figure 1:**
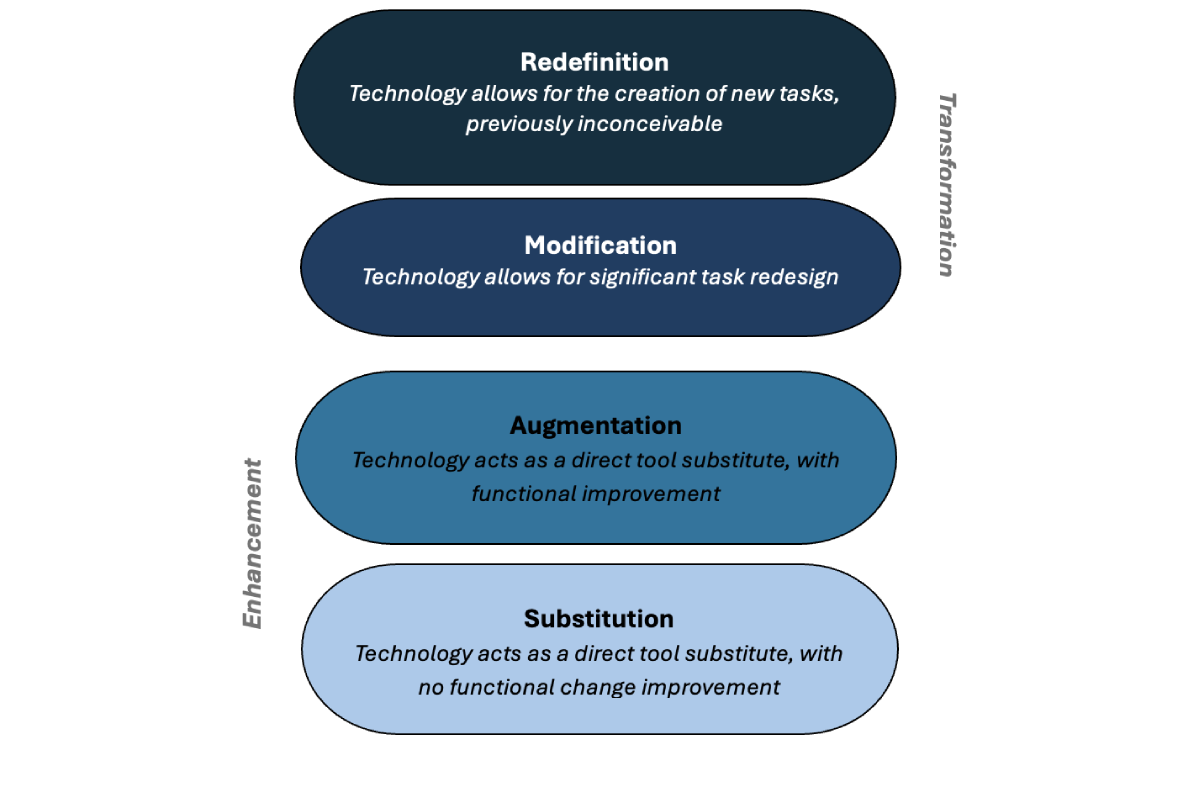
The substitution, augmentation, modification, redefinition (SAMR) model.

The SAMR model underpins our study and serves as an organizing framework for the different types of technology in current use in the institutions under study [34]. The SAMR model is a tool that provides guidance in describing and categorizing uses of educational technology in the classroom. The model is intended to guide educators to enhance their teaching and learning through the adoption, adaptation, or transformation of educational approaches using technology (Figure 1).

To demonstrate our conceptual understanding and application of the SAMR model, we describe each level briefly and include hypothetical examples of each [32,34]. There are 2 dimensions in the model, enhancement and transformation, and 2 levels in each dimension.

1. Enhancement – substitution and augmentation: At the substitution level, digital technology is substituted for analog technology, but the substitution generates no functional change [34,35]. For example, an educator may substitute hard copy handouts with electronic versions. At the augmentation level, technology is exchanged, and the function of the task or tool positively changes in some way. An example is when an educator adds links to electronic versions of learning material. Larger volumes of electronic material, like books and videos, are examples.
2. Transformation – modification and redefinition: At the level of modification, technology enables significant task redesign. Examples of this include the recording of lectures and podcasts, which are textual, visual, or audio tools for the construction of knowledge that can be shared across groups of students. At the redefinition level, technology enables the creation of new tasks that were previously inconceivable, like tools for visualization of narrative and structural aspects of text. Examples of this include artificial intelligence and the use of software for data analysis.

## Methods

### Study Design

This is a descriptive, cross-sectional survey to yield quantitative and qualitative data that address our study aim and objectives outlined below. We aim to assess the needs for TEHPE in higher-education institutions for health professionals in Eastern and Southern Africa.

Relating to the education of health professions in higher education institutions offering health sciences programs, the objectives of the proposed study are as follows:

1. To determine the types of educational technology being used.
2. To identify the frequency with which different technologies are being used.
3. To reveal the reasons why these technologies are being used.
4. To determine teacher and student opinions about the usefulness of the technologies.
5. To identify obstacles to the use of these technologies.
6. To verify factors facilitating the use of these technologies.

### Study Participants

The study population will be health care professionals who are also educators in HPE programs in selected Eastern and Southern African higher institutions of learning. Given the short implementation timeframe for this study, it will be feasible to focus only on educators and students in undergraduate medical and nursing programs in Pan-African Consortium for Technology-Enhanced Health Professions Education (PACoTEH) member countries as the study population. These are higher education institutions offering HPE programs for doctors and nurses at the University of Botswana, National University of Science and Technology (Zimbabwe), University of Zambia, and University of Zimbabwe.

### Inclusion Criteria

Programs in Eastern and Southern African countries that train doctors and nurses, offer bachelor’s degree–level qualifications in medical and nursing programs, are recognized by national professional bodies or government structures, and are offered at accredited tertiary institutions are eligible for inclusion in the study.

### Exclusion Criteria

Undergraduate medical and nursing programs in countries outside of Eastern and Southern Africa and programs whose students are not available during the short period for the study will be excluded.

### Sampling

Due to the nature of the inquiry in our study, nonprobability, purposive sampling will be used to apply our judgment when choosing members of the population expected to possess information on the technology that has been used in HPE [36]. Given the short implementation timeframe due to delays in securing ethics approvals at some institutions, the sample will be drawn from educators and students in undergraduate medical and nursing programs in PACoTEH member countries. Given that these 2 programs are the largest in health professions, the data would be representative of the entire population. Specifically, a program director or educator will be selected by researchers from that institution, drawing on the local knowledge they have of available staff. This person should be fully familiar with the program under consideration. From each program, a minimum of 20 students will be purposively sampled, spread equally over their number of years of study. Educators will also be sampled purposively to obtain a spread of candidates by seniority, gender, and academic discipline.

### Data Collection Procedures

Newly developed questionnaires for staff, students, and educators will be administered using web-based programs (eg, Microsoft Forms) with closed- and open-ended questions to obtain quantitative and qualitative data, respectively. In addition to demographic data, the questions are designed to collect data that answer the study objectives, and some of the questions identify the different types of technology according to the SAMR Model. The questionnaires will be availed to the program director who will identify eligible staff members. The staff members, in turn, will identify students that meet the sample described above. All data will be entered into an electronic database and password-protected. The database will be maintained at one of the consortium institutions. The questionnaires are provided in Multimedia Appendix 1.

### Data Analysis and Presentation

We will build an analytic element to compare data according to the nature of the variables obtained. This will enable us to determine differences in the technology used between programs and countries and the needs for enhancement of the technology. Quantitative data will be summarized using frequency distributions and averages in tables and graphs. Associations and correlations between the data collected for objectives 1-6 (dependent variables) and biographical and institutional data (independent variables) will be determined using relevant statistical techniques. For example, Pearson *r* will be used to measure the correlation between the types of educational technology being used and the frequency across the institutions, and *t* tests and chi-square tests will establish if there is a significant difference, which may lead to the recommendation of some types of technology over others. Qualitative data will be analyzed thematically, and the coding framework will include data familiarization by all authors, display, reduction, concluding and verifying using the study objectives and the SAMR model as a guide to identifying themes [37,38]. The coding will be inductive (generating codes from the data), and while there will be no fixed number of coders, efforts will be made to capture the nuances of the data according to the study objectives. For validation, the authors will check the themes for accuracy and comprehensiveness against the raw data and reach consensus.

Qualitative methodological rigor will be respected to address the transferability, replication, and credibility of our work. Thematic analysis may not lend itself to replicability because it is a form of pattern recognition where themes are identified from the content, requiring inter-rater cross-referencing for reliability and converging meanings from different researchers [39]. The use of the SAMR framework strengthens the trustworthiness of our study because it requires the collection of data that demonstrate the relationship between knowledge and practice. The framework also enables replication through the formatting of data collection methods and analysis. Findings from quantitative and qualitative data analysis will be organized and presented according to the SAMR framework to portray the types of technology in use and challenges encountered. Finally, from the challenges outlined, needs for enhancing technology in HPE will be identified.

### Ethical Considerations

We adhere to relevant safeguards required during research involving human research participants as outlined in the Belmont report of 1979 and the International Conference on Harmonization of 2002 [40,41]. Each institution obtained ethics approval (National University of Science and Technology, NUST/IRB/2024/071, approved April 19, 2024; University of Botswana, UBR/RES/IRB/B10/428, approved September 23, 2024; University of Zambia, 5662-2024, approved August 31, 2024; and University of Zimbabwe, JREC/446/2024, approved October 9, 2024).

To promote confidentiality, identifiable details in the questionnaire will be kept to a minimum. The custodian of the responses will not share this information with other members of the team and will code it before analysis starts. The informed consent procedure will be written in understandable language explaining the aims of the study and the participant’s role in it and list potential risks and benefits, making clear that the participant is free to refuse or withdraw without penalty. The consent form and participant information sheet are given in Multimedia Appendix 2.

### Pilot Study

The instrument (whether validated or not) will be pretested using 2 program directors, 4 students, and 3 educators who will not then be included in the sample. Corrections will be made as indicated. This pilot test will be used to check the clarity of questions and the usability of the web-based format, but no measures of validity are planned.

## Results

This research was funded in October 2023, and approval from the first institutional review board was obtained in April 2024. Data collection commenced in September 2024 and ended in November 2024. Data analysis was accomplished by mid-December 2024. As of August 2025, the manuscript is under peer review for publication of the results.

## Discussion

### Expected Findings

This study aims to assess the needs for enhancing the use of technology in HPE in Eastern and Southern Africa. There has been a movement to promote the use of technology in the education of health professionals worldwide. However, institutions in this region lag behind in the use of technology, and it is not clear where improvements are needed [17,21,23]. The magnitude of the impact of COVID-19 on teaching and learning was more severe in institutions that could not respond to the requirements for remote teaching and learning, and this further exposed the vulnerabilities of the region. To keep up with the pace of industrialization and remain a part of the global village, it is imperative to identify the attributes that enable the use of the variety of technologies that are abundant elsewhere [5].

Data from educators and students is expected to yield quantifiable information on the types of technology available and in use, while qualitative data will reveal experiences of its use. This combination of data, organized along the SAMR framework, informs us on how technology has been substituted and augmented and the modifications and redefinitions that have been applied [32,33,35]. This enables the identification of a variety of practical needs for the improvement of resources and capacity deficiencies that impede the use of technology. We expect this study to glean deeper insights into how to enhance the use of technology in Eastern and Southern Africa.

### Study Limitations

The main study limitation is the small size of the sample, which may not be fully representative of the population. This can be rectified in a subsequent study using stronger probability sampling methods with larger samples or by conducting case studies to give qualitative depth to the main findings. The study is also limited by selection and sampling bias. We choose only to use nursing programs offering a bachelor’s degree in a university. The great majority of nursing programs are at the diploma level, and we will fail to gather information about these relatively under-resourced programs. Additionally, as a multi-institution study, the processes for ethics approval for each institution are beyond the control of the researchers, which may cause delays to the projected timelines.

### Conclusions

The importance of technology in HPE is unquestionable. In this global village, it is imperative to promote uniformity across the education of health professions worldwide. The SAMR framework offers a model to identify gaps, which subsequently enable the institution of measures to bridge them.

## Appendix

Multimedia Appendix 1 Data collection tools.

Multimedia Appendix 2 Consent form and participant information sheet.

## Abbreviations

HPE: health professions education
PACoTEH: Pan-African Consortium for Technology-Enhanced Health Professions Education
SAMR: substitution, augmentation, modification, redefinition
TEHPE: technology-enhanced health professions education

## Data Availability

The datasets generated or analyzed during the current study will be available from the corresponding author upon reasonable request.

Findings will be made available to the funder of the study, to all PACoTEH members, and to all participating programs and institutions. Findings may also be presented at academic conferences and published in peer-reviewed journals.
