# Supplementary material for: Needs for Technology-Enhanced Health Professions Education in Eastern and Southern Africa: Protocol for a Descriptive, Cross-Sectional Survey
Source: JMIR Res Protoc. 2025 Sep 2;14:e67331. doi: 10.2196/67331 (PMC12441635; doi:10.2196/67331)
Supplement: Multimedia Appendix 1 [file resprot_v14i1e67331_app1.docx]

**DATA COLLECTION TOOLS**

**PROGRAM DIRECTOR TOOL**

| Thank you for participating in this study. Please indicate the correct response to each question to the best of your knowledge. Kindly answer all questions in this tool. |
| --- |

**Section A: Demographic characteristics**

A.1 Please select your institution [drop down list of institutions]

A.2 Please select your program [drop down list of programs]

A.3 Please select your qualifications (educational and professional)

A.4 Please select your roles in the institution (education, teaching and learning and specific technology use)

A.5 Do you have any training on the use of technology in education?

A.6 If yes, please specify what you have been trained in regarding technology in education.

**Section B: Types of educational technology**

B.1 Please select all the educational technologies that are used in your program

[A list of all educational technologies]

B.2 Please list all the educational technologies you would require for your program but currently do not have.

**Section C: Frequency of educational technology**

C.1 Please estimate the frequency of the use of educational technologies in your program

| **Educational technology** | **Frequency per week** | **Frequency per month** | **Frequency per semester** |
| --- | --- | --- | --- |
|  |  |  |  |
|  |  |  |  |
|  |  |  |  |
|  |  |  |  |
|  |  |  |  |

**Section D: Reasons for the use of educational technology**

D.1 Please identify the reasons for using educational technology in your program

A. For the creation of new learning tasks which were previously inconceivable.

B. For the re-design of learning tasks.

C. To substitute teaching/ facilitation for improvement of teaching and learning.

D. To substitute teaching /facilitation with no direct improvement on teaching and learning.

E. Other___________ Specify_____________________

D.2 Please list reasons why your academic staff may not be using educational technologies in your institution

**Section E: Opinions regarding the usefulness of educational technology**

Can you describe how useful educational technology has been regarding:

1. The administration of your program
2. The teaching and learning of students in your program.
3. The faculty or educators in your program

**Section F: Obstacles of the use of educational technology**

F.1 Please describe the challenges you face regarding the use of educational technology.

**STUDENT TOOL**

| Thank you for participating in this study. Please indicate the correct response to each question to the best of your knowledge. Kindly answer all questions in this tool. |
| --- |

**Section A: Demographic characteristics**

A.1 Please select your institution [drop down list of institutions]

A.2 Please select your program [drop down list of programs

A.3 Please select your level of study [drop down list of level of study]

**Section B: Types of educational technology**

B.1 Please select all the educational technologies that you have used in your program.

[List of educational technologies]

B.2 Please list all the educational technologies you would require for your program but currently do not have.

**Section C: Frequency of educational technology**

C.1 Please estimate the frequency of the use of educational technologies during your study

| **Educational technology** | **Frequency per week** | **Frequency per month** | **Frequency per semester** |
| --- | --- | --- | --- |
|  |  |  |  |
|  |  |  |  |
|  |  |  |  |
|  |  |  |  |
|  |  |  |  |

**Section D: Reasons for the use of educational technology**

D.1 Please identify the reasons for using educational technology in your program

A. For the creation of new learning tasks which were previously inconceivable.

B. For the re-design of learning tasks.

C. To substitute teaching/ facilitation for improvement of teaching and learning.

D. To substitute teaching /facilitation with no direct improvement on teaching and learning.

D.2 Please list reasons why you may not be using educational technologies in your institution.

**Section E: Opinions regarding the usefulness of educational technology**

Can you describe how useful educational technology has been regarding:

a) The teaching and learning in your program.

**Section F: Obstacles of the use of educational technology**

F.1 Please describe the obstacles you face regarding the use of educational technology in your learning.

_____________________________________________________________________________________

**EDUCATOR TOOL**

| Thank you for participating in this study. Please indicate the correct response to each question to the best of your knowledge. Kindly answer all questions in this tool. |
| --- |

**Section A: Demographic characteristics**

A.1 Please select your institution [drop down list of institutions]

A.2 Please select your program [drop down list of programs]

A.3 Please select the level of students you are involved in teaching

**Section B: Types of educational technology**

B.1 Please select all the educational technologies that you have used in your program.

B.2 Please indicate if you have been trained in the educational technologies that you use in your program

B.3 If yes, please indicate the type of training you have received.

**Section C: Frequency of educational technology**

C.1 Please estimate the frequency of the use of educational technologies during your teaching

| **Educational technology** | **Frequency per week** | **Frequency per month** | **Frequency per semester** |
| --- | --- | --- | --- |
|  |  |  |  |
|  |  |  |  |
|  |  |  |  |
|  |  |  |  |
|  |  |  |  |

**Section D: Reasons for the use of educational technology**

D.1 Please identify the reasons for using educational technology in your program

A. For the creation of new learning tasks which were previously inconceivable.

B. For the re-design of learning tasks.

C. To substitute teaching/ facilitation for improvement of teaching and learning.

D. To substitute teaching /facilitation with no direct improvement on teaching and learning.

D.2 Please indicate the reasons for not using other educational technologies in your program

**Section E: Opinions regarding the usefulness of educational technology**

Can you describe how useful educational technology has been regarding:

1. The teaching and learning of students in your program.
2. The faculty or educators in your program

**Section F: Obstacles of the use of educational technology**

F.1 Please describe the challenges you face regarding the use of educational technology in your learning.
