# Supplementary material for: Needs for Technology-Enhanced Health Professions Education in Eastern and Southern Africa: Protocol for a Descriptive, Cross-Sectional Survey
Source: JMIR Res Protoc. 2025 Sep 2;14:e67331. doi: 10.2196/67331 (PMC12441635; doi:10.2196/67331)
Supplement: Multimedia Appendix 2 [file resprot_v14i1e67331_app2.doc]

Appendix 2: Informed consent form

**INFORMED CONSENT FORM**

**1. Project Title:** Needs assessment for technology enhanced health professions education (HPE) in Eastern and Southern Africa

**2. Principal Investigator:** Dr Shalote Chipamaunga

**3. Co-Investigators:** Dr Rudo Gwini

Dr Detlef Prozesky

Dr Elliot Kafumukache

Prof Patricia Katowa-Mukwato

Dr Mike Nantamu Kagawa

Prof Lynette Jean van der Merwe

Prof Kefalotse Dithole

Prof Champion Nestai Nyoni,

**4. PURPOSE OF STUDY:** The purpose of this study is to assess the needs for technology enhanced HPE in Eastern and Southern Africa.

**5. PROCEDURES AND DURATION:**

This is a descriptive, cross-sectional survey. The model Substitution, Augmentation, Modification, Redefinition (SAMR) underpins our study and serves as an organizing framework for the different types of technology in current use in the institutions under study. Using an online questionnaire, data will be collected from medical and nursing programmes at Bachelor’s degree level recognized by national professional bodies and/or government structures offered at tertiary institutions in Eastern and Southern Africa countries.

**6. RISKS AND DISCOMFORTS**:

We do not anticipate any risks and discomforts for the participants because of the study. The study simply aims to determine needs for technology in health professions education.

**7. BENEFITS AND/OR COMPENSATION:**

Overall, the study will provide clear information about the needs for enhancing technology use in health professions education. It is likely that different technology will have been applied from country to country and programme to programme, thereby benefiting:

- Teachers and administrators in the programmes being investigated, by making them aware of the technology that others have developed and thereby broadening their options for dealing with the technology challenges they face.
- Subjects who provide information for the research, by being beneficiaries of improved online teaching/ learning programmes in their own institutions.
- Teachers and administrators in other programmes, again by being informed of technology that others have used and thereby gaining insight into other options for their own programmes.

**8. CONFIDENTIALITY:**

We will include the following information: If you choose to take part in the study, any information including personal details regarding your gender, age, names or initials, and opinions will be processed anonymously into any research report. Your name will not be written on the questionnaire you complete. Only a document identity number (code) will appear in the report. All data will be entered into an electronic password-protected database kept at one of the investigators’ institutions’ data base. The PI will make this data available upon written request.

Future use of information

The results of this study will be shared in the form a report with the Deans responsible for all the programmes taking part in the research. The Deans may also decide to share the information with other relevant stakeholders in each programme. Findings will also be published at local, regional and international forums and by publications in medical journals.

**9. IN THE EVENT OF INJURY:**

We do not anticipate any injury to the participants because of the study. The study simply aims to determine needs for technology in health professions education.

**10. VOLUNTARY PARTICIPATION:**

We will include the following statement: Your participation in the study is voluntary. You may choose to withdraw consent and participation in the study at any stage without prejudice. This is further affirmed by giving their signed authorization in Item 12 below.

**11. OFFER TO ANSWER QUESTIONS**

Before you sign this form, please ask any questions on any aspect of this study that is unclear to you. You may take as much time as necessary to think it over.

**12. AUTHORIZATION**

YOU ARE MAKING A DECISION WHETHER OR NOT TO PARTICIPATE IN THIS STUDY. YOUR SIGNATURE INDICATES THAT YOU HAVE READ AND UNDERSTOOD THE INFORMATION PROVIDED ABOVE, HAVE HAD ALL YOUR QUESTIONS ANSWERED, AND HAVE DECIDED TO PARTICIPATE.

| 1. | I have read and understood the information about the project, as provided in the Information Sheet. |  |
| --- | --- | --- |
| 2. | I have been given the opportunity to ask questions about the project and my participation. |  |
| 3. | I voluntarily agree to participate in the project. |  |
| 4. | I understand I can withdraw at any time without giving reasons and that I will not be penalized for withdrawing nor will I be questioned on why I have withdrawn. |  |
| 5. | The procedures regarding confidentiality have been clearly explained (e.g. use of names, etc.) to me. |  |
| 6. | The use of the data in research, publications, sharing and archiving has been explained to me. |  |
| 8. | I understand that other researchers will have access to this data only if they agree to preserve the confidentiality of the data and if they agree to the terms I have specified in this form. |  |
| 9. | I am aged 18 years or older. |  |

**Participant:**

………………………… ……………………… ………………

Name of Participant Signature Date

**Researcher:**

………………………… ……………………… ………………

Name of Researcher Signature Date
